# Resveratrol Loaded Liposomes Disrupt Cancer Associated Fibroblast Communications within the Tumor Microenvironment to Inhibit Colorectal Cancer Aggressiveness

**DOI:** 10.3390/nano13010107

**Published:** 2022-12-25

**Authors:** Paweena Dana, Nutthanit Thumrongsiri, Prattana Tanyapanyachon, Walailuk Chonniyom, Primana Punnakitikashem, Nattika Saengkrit

**Affiliations:** 1 National Nanotechnology Center (NANOTEC), National Science and Technology Development Agency (NSTDA), Pathum Thani 12120, Thailand; 2 Department of Biochemistry, Faculty of Medicine Siriraj Hospital, Mahidol University, 2 Wanglang Road, Bangkoknoi, Bangkok 10700, Thailand; 3 Research Network NANOTEC-Mahidol University in Theranostic Nanomedicine, Faculty of Medicine Siriraj Hospital, Mahidol University, 2 Wanglang Road, Bangkoknoi, Bangkok 10700, Thailand

**Keywords:** resveratrol, colorectal cancer, cancer associated fibroblast, metastasis, drug sensitivity, liposome

## Abstract

Colorectal cancer (CRC) is a cancer-associated fibroblast, CAF-rich tumor. CAF promotes cancer cell proliferation, metastasis, drug resistance via secretes soluble factors, and extracellular matrices which leads to dense stroma, a major barrier for drug delivery. Resveratrol (RES) is a polyphenolic compound, has several pharmacologic functions including anti-inflammation and anticancer effects. Considering tumor microenvironment of CRC, resveratrol-loaded liposome (L-RES) was synthesized and employed to inhibit CAF functions. The L-RES was synthesized by thin-film hydration method. The cytotoxicity of L-RES was evaluated using MTT assay. Effect of L-RES treated CAF on tumor spheroid growth was performed. Cell invasion was determined using spheroid invasion assay. The effect of L-RES on 5-fluorouracil (5-FU) sensitivity of CRC cells was determined in co-cultured tumor spheroids. Subtoxic dose of L-RES was selected to study possible inhibiting CAF functions. Decreased CAF markers, α-SMA and IL-6 levels, were observed in L-RES treated activated fibroblast. Interestingly, the activated fibroblast promoted invasive ability and drug resistance of CRC cells in co-culture condition of both 2D and 3D cultures and was attenuated by L-RES treatment in the activated fibroblast. Therefore, L-RES provides a promising drug delivery strategy for CRC treatment by disrupting the crosstalk between CRC cells and CAF.

## 1. Introduction

Colorectal cancer (CRC) is a malignancy of the colon and rectum. CRC is the third most common cancer type worldwide [1,2]. Liver metastasis and tumor recurrence are two main problems resulting in poor prognosis of CRC patients [1,3]. Hence, research that focuses on inhibiting tumor metastasis of CRC is urgently needed to improve survival of CRC patients.

In solid tumors, a dense accumulation of fibrosis and cellular stroma, namely the desmoplastic reaction, is recognized as a prominent feature of pancreatic cancer, prostate cancer, breast cancer, cholangiocarcinoma, and of CRC. The stromal compartment in the solid tumor microenvironment consists of cancer-associated fibroblasts (CAF), endothelial cells, inflammatory cells, and extracellular matrix (ECM). The tumor microenvironment (TME) promotes tumor progression, metastasis, and resistance to therapeutics, such as chemotherapy and radiotherapy [4,5,6]. In the TME, CAF is a majority component that plays a critical role in promoting tumor development [7,8]. A quiescent or resting fibroblast can be activated in response to stimulants, such as growth factors, cytokines, and stress, to become an activated fibroblast [8,9,10]. Transforming growth factor beta 1 (TGF-β1), secreted by stromal and tumor cells, enhances the transition of fibroblasts into CAF, resulting in tumor migration and metastasis. The expressions of traditional biomarkers, such as smooth-muscle actin (α-SMA), fibroblast activation protein (FAP), interleukin-6 (IL-6), monocyte chemoattractant protein-1 (MCP-1), and platelet-derived growth factor (PDGF) receptor, are employed to identify CAF [8]. In the tissues of CRC patients, accumulation of CAF was assessed by immunohistochemical analysis using stromal FAP, a CAF marker. The patients who had high expression of FAP were correlated with an aggressive phenotype of cancer, metastasis, and shorter survival than patients with low FAP expression [11].

In the tumor microenvironment, CAF play several roles in promoting tumor growth and tumor metastasis through the secretion of growth factor and cytokines, chemokines, and ECM protein. These signals help activate numerous signaling pathways involved in cell proliferation and metastasis, which result in poor patient outcomes [4,8,12]. During the progression of cancer, CAF contribute by the mechanical remodeling of the stromal ECM. CAF secretes several ECM proteins that lead to dense stroma and high interstitial fluid pressure. Furthermore, the alteration of the matrix architecture in solid tumors significantly impairs chemotherapeutic delivery to tumor cells [13,14].

As the main component in the TME, CAF is recognized as a target site for cancer therapy. This approach is based on reprogramming or normalizing the microenvironment, which consequently reduces the physiological barriers for drug delivery [13,14,15,16,17,18]. Both the eradicating and reprogramming of CAF have been studied, and this strategy focuses on depleting CAF in cancer treatment. However, the results revealed systemic side effects in mice [19]. Therefore, at present, reprogramming CAF function or deprogramming activated fibroblasts are more attractive strategies for developing cancer therapeutics [20]. When using CAF for targeted cancer therapy, the monoclonal anti-bodies and small molecule drugs that target crucial regulatory factors have been reported in both pre-clinical and clinical studies [21,22]. The formidable barrier caused by CAF function is the crucial factor impeding drug delivery. The disruption of the barrier, which caused by CAF, is the one of interesting strategy for enhancing drug delivery to achieve cancer treatment. Therefore, we can design nanoparticles to diminish CAF activation. Therefore, we can design nanoparticles to target CAF. The nanoparticle targeting CAF could reshape the TME or CAF and enhance drug delivery efficiency into tumor cells. Currently, several CAF-targeting nanoparticles have been reported that accomplished antitumor effects by eradicating CAF or reshaping the CAF functions [23,24,25].

Resveratrol (3,5,4’-trihydroxystilbene; RES) is a natural polyphenol and phytoalexin conferring therapeutic activities, such as antioxidant, anti-inflammatory, and anticancer properties [26,27,28]. However, RES has limited clinical utility due to its poor water solubility, low chemical stability, and short biological half-life [29]. In the current decade, several studies have used RES as both an adjunct therapy and a chemopreventive dietary supplement for cancer treatments. In addition, the anticancer activity of resveratrol is reportedly enhanced when used as a combination therapy with other chemotherapeutic drugs [30,31,32,33]. RES had been used to interrupt crosstalk between CAF and cholangiocarcinoma cells and thus inhibit cell invasion of the cholangiocarcinoma cells [34]. Numerous studies have reported that resveratrol inhibits cell proliferation of cancer cells and tumor growth in vitro and in vivo in breast, colon, prostate, and lung cancers [24,35,36]. Unfortunately, clinical trials showed unsatisfactory results with no effect on tumor growth [28]. This phenomenon may be due to the low aqueous solubility, chemical instability, and poor bioavailability of RES in human patients, which limit using RES as a chemopreventive or therapeutic agent. To overcome these limitations, nanoparticle-based formulations of resveratrol have been developed to enhance its absorption and deliver the optimal concentrations of resveratrol to the target tumor tissue. 

This novel nanomedicine approach can increase water solubility, stability, and permeation of drugs across biological membranes. Nanomedicine provides an enhanced permeation and retention effect (EPR) which certain sizes of nanoparticle can accumulate at the tumor much more than in normal tissue according to angiogenesis and leakage of blood vessel in tumor tissue [37]. The nanocarrier is required to deliver resveratrol, which is a very high hydrophobic drug. Furthermore, nano-based lipid carrier is biocompatible for cells and organs as well. Liposome was employed for delivering resveratrol by prolong the drug at stroma. Therefore, this study aimed to develop the resveratrol-loaded liposome (L-RES) for improving the therapeutic effect of RES. RES was used to interrupt crosstalk communication between activated fibroblasts in the tumor microenvironment and in colorectal cancer cells to inhibit the aggressiveness of the colorectal cancer cells. L-RES-inhibited activated fibroblasts was investigated in a co-cultured model tumor-promoting activity of activated fibroblasts and chemosensitizing ability of L-RES were studied.

## 2. Materials and Methods

### 2.1. Materials

Dulbecco’s Modified Eagle Medium (DMEM) (Cat. No. 12800017), Penicillin-Streptomycin-Glutamine (100X) (Cat. No. 10378016), trypsin-EDTA (Cat. No. 25200056), fetal bovine serum (FBS) (Cat. No. 10270106), and Interleukin-6 (IL-6) enzyme-linked immunosorbent assay (ELISA) (Cat. No. 88-7066-22) were purchased from GIBCO Invitrogen (Grand Island, NY, USA). Matrigel Growth Factor Reduced (Cat. No. 354230) 96 well plate (Cat. No. 3590), 96 well round-bottom ultra-low attachment plates (ULA) (Cat. No. 7007) and 8.0 μm pore size insert (Cat. No. 3422) were purchased from Corning Inc. (Corning, NY, USA). MTT assay, 3-(4,5-dimethylthiazol-2-yl)-2,5-diphenyltetrazolium bromide (Cat. No. 475989) was purchased from EDM Millipore Corp. (Burlington, MA, USA). Resveratrol (Cat. No. R5010) and dimethyl sulfoxide (DMSO) (Cat. No. 102952) were purchased from Merck Millipore (Sigma Aldrich; Merck Millipore, Darmstadt, Germany). Soybean lecithin (soya phosphatidylcholine; PC) was purchased from Degussa (Hamburg, Germany). Cholesterol was purchased from Wako Fujifilm (Osaka, Japan). Sulforhodamine B (Cat. No. S1402-1G) was purchased from Sigma-Aldrich (Darmstadt, Germany). Two hundred mesh carbon-coated copper grids (Cat. No. EMS200-Cu) were purchased from Electron Microscopy Sciences (Hatfield, PA, USA). A dialysis bag with a molecular weight cut-off of 100 kDa was purchased from CelluSep (Seguin, TX, USA). TRIzol reagent (cat. #15596026) was purchased from Thermo Fisher Scientific (Thermo Fisher Scientific, Waltham, MA, USA). iScript Reverse Transcription Kits (cat. #1708840) was purchased from Bio-Rad Laboratories, Hercules, CA, USA. Luna Universal qPCR (cat. #M3003L cat. #) was purchased from New England Biolab Inc., Ipswich, MA, USA.

### 2.2. Methods

#### 2.2.1. Cell Culture

The human colorectal adenocarcinoma cell line, HT-29 and human lung fibroblast cell line, MRC-5, were purchased from the American Type Culture Collection (ATCC HTB-38 and ATCC CCL-171, respectively, Manassas, VA, USA) were purchased from the American Type Culture Collection (ATCC number HTB-37 and HTB-38, respectively). Cells were cultured in DMEM medium supplemented with 10% FBS, 0.1 mM non-essential amino acids (100 µg/mL L-glutamine), 100 μg/mL streptomycin, and 100 U/mL penicillin). Cells were cultured in an incubator at 37 °C with a humidified atmosphere containing 5% CO_2_.

#### 2.2.2. Formulation of Resveratrol-Loaded Liposome (L-RES)

To prepare the L-RES, a conventional thin film method was used. Mixtures of soybean lecithin and cholesterol with molar ratio 8:1, and 0.1% Phosphatidyl Ethanolamine - Polyethylene Glycol (PE-PEG) were prepared. The mixture was dissolved in chloroform-methanol (2:1 *v*/*v*, 10 mL). Resveratrol (10 mg) was dissolved in 2 mL of methanol and added to the mixture. The solvent was then removed by rotary evaporation at 25 °C under 50–100 kg/cm^2^ nitrogen flow to obtain a thin lipid film. After air drying, the thin lipid film was rehydrated with 10 mL of phosphate-buffered saline (PBS), pH 7.4, by shaking it on an incubator shaker at room temperature (RT). To prepare vesicles, the liposomes were extruded for 15 passages through a mini extruder (Avanti Polar Lipids, Alabaster, AL), using Whatman 200 nm polycarbonate membranes (GE Life Sciences, Pittsburgh, PA). The unencapsulated RES was removed by centrifugation (To-myMX-301, Tokyo, Japan) at 80,000 rpm for 1.5 h. To gain the L-RES, the pellet was re-suspended in a PBS buffer (pH 7.4).

#### 2.2.3. Physicochemical Characterization of L-RES

The morphological characteristics of empty liposome (LIP) and L-RES were observed under transmission electron microscopy (TEM). Negative staining technique was used to visualize the samples. Briefly, 5 µL of sample was dropped on a 200 mesh carbon-coated copper grid for 10 min. After that, the excess of sample was removed by filter paper absorption, followed by air-dry. Then, the prepared grid was subjected to TEM (model JEM 2010; JEOL, Peabody, MA). The sample was photograph with 80 kV at the magnification of 25,000×, 50,000×, and 80,000×, respectively.

The size, polydispersity index (PDI) and zeta potential of LIP and L-RES were measured by dynamic light scattering (DLS) (Zetasizer; nanoseries, Malvern; Worcestershire, UK). To avoid viscosity effects, 20 µL of sample was diluted with 1 mL of filtered distilled water. Measurements were performed in triplicate at 25 °C. 

#### 2.2.4. In Vitro Drug Release Study of L-RES

The releasing profile of resveratrol was determined using the dialysis bag method. The liposome solution (0.5 mL) was loaded into the dialysis bag with a molecular weight cut-off of 100 kDa. The solution was dialyzed against a PBS buffer, pH 7.4, with 1.5% TWEEN 80 (15 mL) at 37 °C, 180 rpm shaking [38]. One milliliter of PBS buffer was collected and replaced with equal volume at 0, 1, 2, 4, 6, 24, and 48 h during the dialysis process. The concentration of RES in the collected medium was quantified using high-performance liquid chromatography (HPLC) following the protocol described elsewhere [38]. Briefly, the mixed solvent of methanol and water (65:35) was used as a mobile phase in a C18 column. A detection wavelength of 305 nm was used to detect resveratrol.

#### 2.2.5. MTT Assay for Cytotoxicity Evaluation of L-RES

The cytotoxicity of L-RES was determined using the MTT tetrazolium reduction assay. MRC-5 and HT-29 cell lines were independently plated into a 96 well plate at the concentration of 5 × 10^3^ and 8 × 10^3^ cells/well, respectively, in 100 µL DMEM complete medium and cultured overnight at 37 °C in a humidified atmosphere with 5% CO2. Cells were treated with either free resveratrol (RES) or LIP or L-RES at various concentrations of RES at 0–500 µM in 10% FBS contained media. Then, 10 µL of MTT stock solution (5 mg/mL) was added to each well 24 h. following treatment. The medium was removed after 4 h of incubation and formazan crystals were completely dissolved by adding 100 µL of DMSO. Absorption values at wavelengths 570 nm were measured by using a microplate reader (Spectra-MAX, Molecular Devices, Poway, CA, USA). Each experiment was independently conducted in triplicates. Three independent experiments were performed.

#### 2.2.6. Activation of Fibroblast Using CRC-Derived Conditioned Medium

To mimic the conditions of a cancer-associated fibroblast (CAF), a normal human fibroblast was activated to become an activated fibroblast (AFB) using HT-29-derived conditioned medium (CM). Briefly, MRC-5 normal lung fibroblast cells (2 × 10^5^) were seeded into a 6-well plate and cultured overnight. Cells were replaced medium with 50% HT-29 CM in DMEM complete medium for 24 h. After 24 h of incubation, the medium was replaced with fresh serum-free DMEM medium and incubated for another 24 h. The medium was collected for further analyses, such as for levels of interleukin-6 (IL-6), and activities of matrix metalloproteinases 2 and 9 (MMPs-2/-9). Since the expression of α-smooth muscle actin (α-SMA), a well-known marker of activated fibroblast, is increased expression in CAF compared to normal fibroblast [8]. Overexpression of α-SMA is one of distinct character between CAF and normal fibroblast. Then, the α-SMA was used as a CAF marker to confirm the activated fibroblast after inducing by HT-29 CM in this study. In addition, CAF exhibited higher levels of IL-6 and MMPs related with increased invasive ability compared with normal fibroblast [6,39,40]. To confirm activation of fibroblast after inducing with HT-29 CM, the expression of α-SMA, levels of IL-6 and activities of MMPs were used as markers of activated fibroblast. Cells were labelled with the activated fibroblast marker α-smooth muscle actin (α-SMA) using immunofluorescent labelling method. The level of IL-6 was determined using enzyme-linked immunosorbent assay (ELISA) following the manufacturer’s instructions. Activities of MMPs-2/-9 were assessed using gelatin zymography assay following the protocol described elsewhere [41]. 

#### 2.2.7. Inactivation of Activated Fibroblast Using L-RES

To test the effect of L-RES on inactivating the activated fibroblast, MRC-5 cells were activated as Act-MRC-5, as described in Section 2.2.6. The Act-MRC-5 cells were treated with free RES, LIP, or L-RES at concentration of 25 and 50 µM for 24 h. After incubation, the media were replaced with serum-free media for 24 h in order to avoid the effect of the remained RES, LIP or L-RES. The Act-MRC-5 CM was collected to determine the IL-6 levels using ELISA. To evaluate the expression of α-SMA, the Act-MRC-5 cells were labelled via immunofluorescent staining against α-SMA.

#### 2.2.8. Effect of L-RES-Treated Act-MRC-5 on 3D Tumor Spheroid Cell Viability of CRC Cells

The effect of L-RES-nanoparticle-treated Act-MRC-5 on CRC cell growth in indirect co-culture model was tested on 3D spheroid cultures. HT-29 cells (1,500 cells/well) in 100 µL of culture medium were seeded into 96 well round bottom ultra-low attachment plates (ULA) and incubated for 3 days to form spheroids. Afterward, 100 µL of each sample of Act-MRC-5-derived CM (RES-, LIP-, and L-RES-treated Act-MRC-5 CM) was added to the tumor spheroids and incubated for a further 2 days. Bright-field images of HT-29 tumor spheroids treated with the samples were taken with an Olympus IX50 inverted microscope (Olympus, Tokyo, Japan). 

To investigate the effect of L-RES-treated MCR-5* cells on the chemotherapeutic drug sensitivity of CRC cells, a combination of Act-MRC-5-derived CM (RES-, LIP-, and L-RES-treated Act-MRC-5 CM) and the chemotherapeutic drug 5-fluorouracil (5-FU) was administered in the tumor spheroid. Three-day-old HT-29 spheroids were concomitantly treated with Act-MRC-5-derived CM (RES-, LIP-, and L-RES-treated Act-MRC-5 CM) and combined with 5-FU at a concentration of 5 µM. After 48 h of incubation, the HT-29 tumor spheroid morphology was observed. The spheroid volume was quantitated using ImageJ software (Bethesda, MD, USA). The experiment was performed in triplicated wells.

#### 2.2.9. Cell Invasion

Cell invasion of the HT-29 cell line co-cultured with activated fibroblast Act -MRC-5 cells was analyzed using the Boyden chamber assay with 8.0 μm pore size insert and a Matrigel coated (0.4 mg/mL) insert for invasion assay. The MCR-5 cells (2 × 10^4^ cells) were seeded into the lower chamber and cultured overnight. Afterward, the HT-29-derived CM was added to the MRC-5 cells induced into Act-MRC-5 for 24 h of incubation; next, the RES, LIP, and L-RES were added to the Act-MRC-5 and cultured for 24 h. The next day, the HT-29 cell suspension (6 × 10^4^ cells) in serum-free medium were seeded into the insert and put on the lower chamber containing Act-MRC-5 and were allowed to migrate or invade for 24 h. Cells on the upper surface of the insert were removed by scraping with a cotton bud, and cells on the lower surface of the insert were fixed and stained with sulforhodamine B (Cat. No. S1402-1G, Sigma-Aldrich, Darmstadt, Germany). Migrated or invaded cells were counted under a microscope (five microscopic fields/well). Each experiment was performed in triplicates. Two independent experiments were performed.

#### 2.2.10. Spheroid Invasion Assay

Three-day-old spheroids were harvested and embedded on diluted Matrigel 1:32 (cold serum-free DMEM medium) in ultra-low attachment plate in volume of 100 µL. The plate was then immediately put in the incubator to let the Matrigel solidify. After one h, the Act-MRC-5-derived CM (RES-, LIP-, and L-RES-treated Act-MRC-5 CM) was added to the embedded spheroid. The complete media was used as a control. The HT-29 tumor spheroids were captured at times of 0, 24, 48, 72, and 96 h using an Olympus IX50 inverted microscope (Olympus, Tokyo, Japan). The spheroid-invaded area was quantitated using ImageJ software. The experiment was performed in triplicated wells.

#### 2.2.11. Quantitative Real-Time PCR

To explore the molecular mechanism underlying diminished activation of fibroblast by L-Res enhances 5-FU sensitizing of CRC cells, total RNA was extracted using TRIzol reagent. cDNA was then prepared using iScript Reverse Transcription Kits. Quantitative Real-Time PCR analysis was performed using the Luna Universal qPCR. The mRNA expression levels of epithelial to mesenchymal transition (EMT) related genes, namely Slug, Vimentin, E-cadherin, and N-cadherin were normalized with Ct of β2Mg and calculated as ∆Ct = Ct target − Ct β2Mg. The 2^−∆∆Ct^ was used to calculate the fold change. The oligonucleotide primers of Vimentin, E-cadherin, and N-cadherin used were previously reported [42,43].

#### 2.2.12. Statistical Analysis

The data are expressed as the mean ± standard deviation. The significant differences observed between the experimental groups were determined using Student’s *t*-test. *p* < 0.05 was considered to indicate a statistically significant result. All statistical analyses were performed using SPSS 17.0 (SPSS Inc., Chicago, IL, USA).

## 3. Results and Discussion

### 3.1. Morphology and Physicochemical Characteristics of L-RES

**Figure 1:**
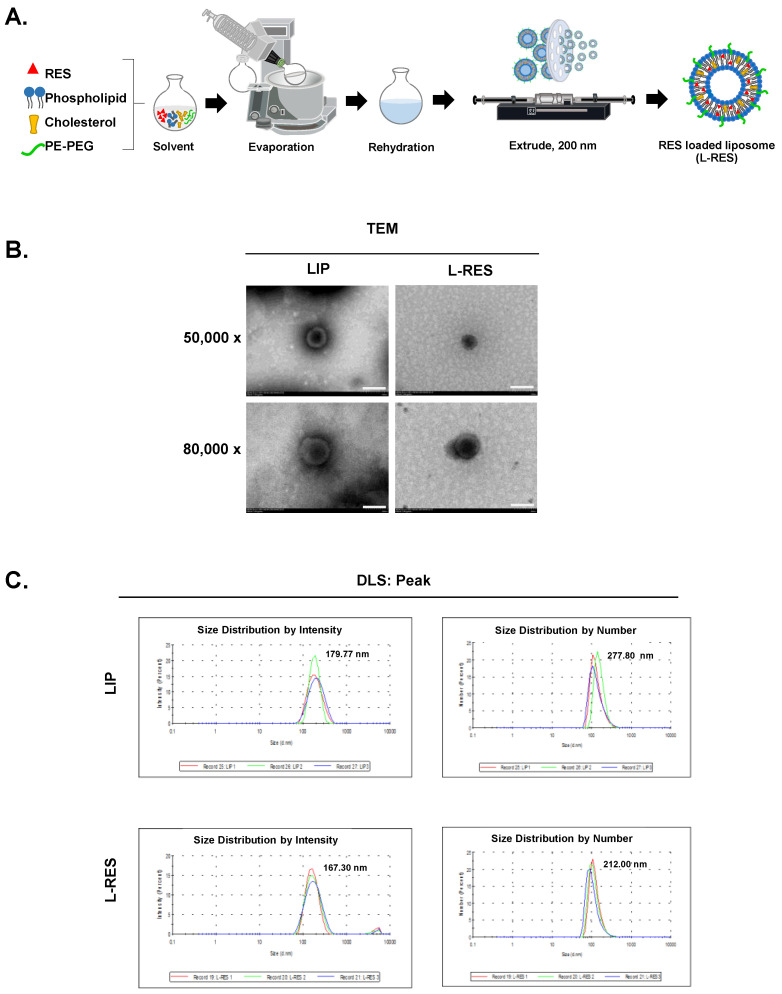
Formulation and morphology of resveratrol-loaded liposome (L-RES). (**A**) The L-RES was formulated using the thin film method. (**B**) Morphology of the formulated L-RES was observed under transmission electron microscopy (TEM) with magnifications 50,000× and 80,000×; scale bar = 200 nm. (**C**) Peak of average diameter of L-RES as determined by dynamic light scattering (DLS).

The L-RES was prepared using the thin film hydration method with the lipid components, as shown in Figure 1A. Transmission electron microscope (TEM) was employed to observe the sizes and morphologies of the LIP and L-RES. LIP and L-RES exhibited a spherical shape with a diameter of approximately 170–190 nm, as observed under magnifications 50,000× and 80,000× (Figure 1B). The TEM images indicated that the diameters of LIP and L-RES were 100–200 nm, which are preferentially extravasated into the tumor tissues via the EPR effect [44]. The physicochemical characteristics of empty liposome (LIP) and L-RES were determined. As indicated in Table 1, the size diameters and zeta potentials of LIP and L-RES were evaluated using DLS. The average diameters of the LIP and L-RES nanoparticles were 179.77 ± 5.67 nm and 167.30 ± 2.79 nm, respectively. The average zeta potential of LIP and L-RES nanoparticles were −11.33 ± 0.38 mV and −10.90 ± 0.46 mV, respectively. Both LIP and L-RES were shown to be homogeneous, as demonstrated by a narrow size distribution with a PDI value of 0.11 ± 0.03 and 0.20 ± 0.02, respectively. The single peaks of average diameters of the LIP and L-RES nanoparticles were also detected (Figure 1C). This result agrees well with the low PDI of liposome nanoparticles (<0.2), and thus indicates that the liposomes have narrow size distributions [45].

### 3.2. Drug Release Study of L-RES

**Figure 2:**
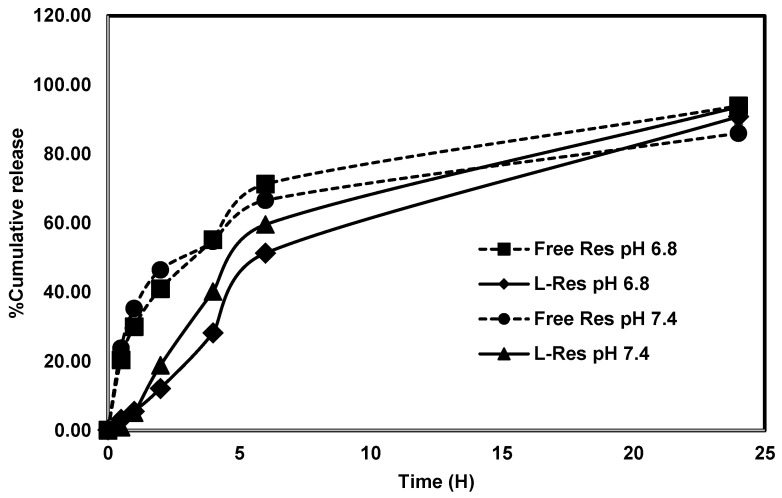
Drug release profile of L-RES. (A) Drug release profiles were assessed using a dialysis bag, molecular weight cut-off of 100 kDa, in phosphate-buffered saline (PBS), pH 7.4, with 1.5% TWEEN 80 at various time points (0–24 h). Data are presented as mean ± SD. The graph represents data from two independent experiments. *p* < 0.05, Student’s *t*-test.

The drug release profiles of L-RES are shown in Figure 2. The conditions for the drug release study of L-RES were determined from the equilibrium solution of resveratrol in PBS (0.01 M) containing 0.2% TWEEN 80 at pH 6.8 and pH 7.4. The release of RES from L-RES was significantly prolonged compared with free RES at pH 6.8 and pH 7.4. At pH 6.8, which mimics the acidic condition in the tumor environment [46], the cumulative drug release from L-RES was 12.20 ± 0.09%, 28.28 ± 0.31%, and 51.33 ± 0.73%, respectively, while free RES was 41.01 ± 0.68%, 55.25 ± 0.97%, and 71.31 ± 1.0% at 2, 4, and 6 h, respectively. At the end of the experiment at pH 6.8, cumulative drug release from L-RES was 90.83 ± 2.22%, while free RES was 93.94 ± 1.63%. For the condition at pH 7.4, which mimics the physiological environment, cumulative drug release from L-RES was 18.85 ± 0.39%, 40.20 ± 0.41%, and 59.61 ± 0.12%, while free RES was 46.47 ± 1.17%, 54.67 ± 0.78%, and 66.58 ± 0.97% at 2, 4, and 6 h, respectively. By the end of the experiment at pH 7.4, cumulative drug release from L-RES was 93.68 ± 0.45%, while free RES was 86.01 ± 1.52%. Compared with free RES, RES in L-RES was slowed at both pH 6.8 and pH 7.4, which revealed that encapsulation of RES could enhance the prolonged release profile. However, the prolonged release at pH 6.8 was considerably better than at the pH 7.4 condition. This result indicates that the formulated L-RES is beneficial in acidic conditions in the tumor’s microenvironment compartment [46]. Therefore, the formulated L-RES could be used as a nanocarrier to specifically target the tumor site at a specific pH to release RES.

### 3.3. CRC-Derived CM-Induced Activation of Fibroblasts

To mimic the characteristics of CAF, MRC-5 normal human lung fibroblast cells were induced into activated fibroblasts (Act-MRC-5) using CRC-derived CM, as described in the Methods section. The α-SMA was used as a well-known CAF or activated marker [8]. Expressions of α-SMA were determined in Act-MRC-5 cells compared with the normal MRC-5 cells using immunofluorescent staining. The expression of α-SMA was increased in the Act-MRC-5 cells compared with MRC-5 cells (Figure S1A). The IL-6 levels and MMPs-2/-9 activities were determined in the activated MRC-5 (Act-MRC-5) CM using ELISA and gelatin zymography assays, respectively. Compared with those of normal MRC-5, the IL-6 levels of Act-MRC-5were elevated from 131.5 pg/mL ± 0.1 to 4073.3 pg/mL ± 2.5 (Figure S1B). This result corresponds with the results of previous studies, in which the activated fibroblast had higher levels of IL-6 than the normal fibroblast [6,39,40]. Increased activities of MMPs have been reported in CAF or activated fibroblasts compared with normal fibroblasts [47,48]. In this study, the gelatin zymography revealed that the activities of MMPs-2/-9 in Act-MRC-5 were increased compared with normal MRC-5 CM (Figure S1C). Furthermore, the cell migration and invasion abilities of Act-MRC-5were increased compared with inactivated MRC-5 cells (Figure S1D). These results emphasize that the CRC-derived CM could successfully induce fibroblast activation of MRC-5 cells.

### 3.4. Evaluation of Cytotoxicity of L-RES in Fibroblast and CRC Cell Lines

**Figure 3:**
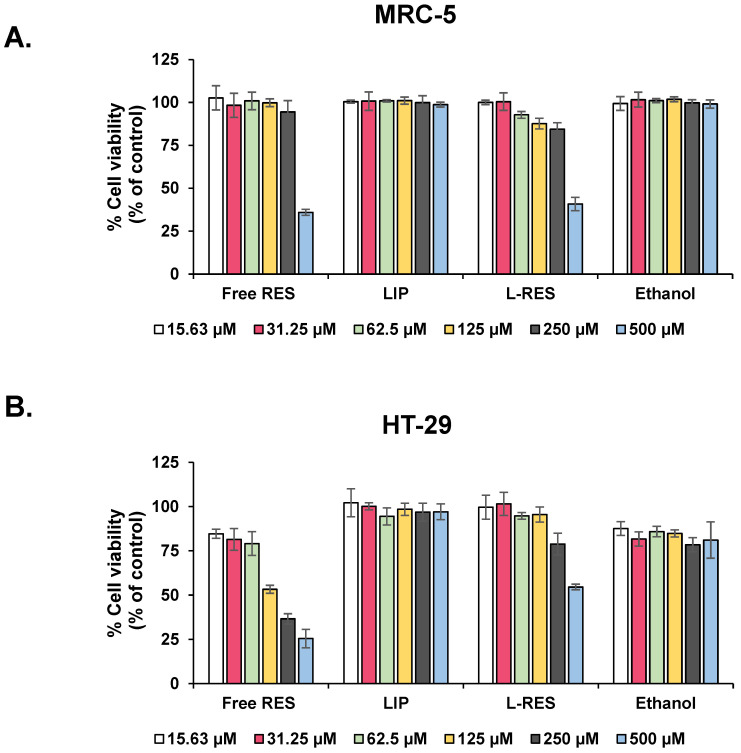
Cytotoxicity of RES, LIP, and L-RES against colorectal cancer and normal human fibroblast cell lines. (**A**) Cell viability of MRC-5 cells. (**B**) HT-29 cells in monolayer were exposed to RES, LIP, and L-RES for 24 h. MTT assay was used to analyze cell viability. Data are shown as representative data of two independent experiments, and error bars represent SD.

The cytotoxicity of L-RES in fibroblasts was determined using MTT assay. A sub-toxic dose lower than IC_20_ was selected for further study in terms of its inactivation of activated fibroblast (Figure 3A). The concentration of L-RES at 62.5 µM and concentrations lower than 62.5 µM had cell viability higher than 80%, of which 89.75 ± 5.5 µM were selected. At the same concentration, free RES exhibited cell viability of fibroblasts at 100.11 ± 5.1%, while that of empty liposomes was 101.91 ± 0.6%.

For the cytotoxicity of L-RES in CRC cells, the L-RES and free RES reduced cell viability of HT-29 cells in a dose-dependent manner, as shown in Figure 3B. In HT-29 cells, at a concentration of 62.5 µM, cell viability of cells treated with L-RES, free RES, and LIP were 94.7 ± 1.8%, 79.1 ± 6.7%, and 100.1 ± 2.0%, respectively.

### 3.5. Inactivation of the Activated Fibroblast by L-RES

**Figure 4:**
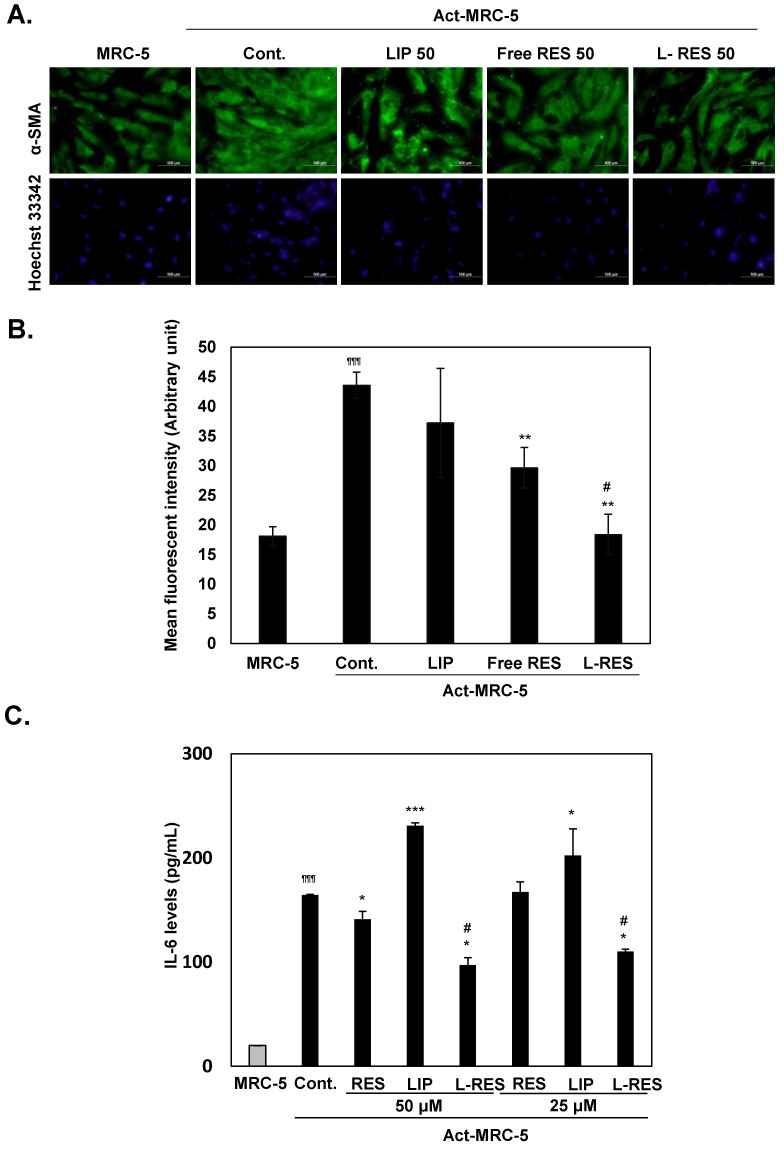
L-RES could inhibit activated fibroblast. The MRC-5 cells were activated with HT-29 cells derived in conditioned medium to become Act-MRC-5 activated fibroblasts. The Act-MRC-5 were then treated with RES, LIP, and L-RES at concentrations of 50 µM for 24 h. (**A**) Expression of α-SMA in Act-MRC-5-treated cells was determined by immunofluorescent staining. (**B**) Quantitative analysis of α-SMA expression. The quantitative data was analyzed from three different fields observing under microscope. (**C**) IL-6 levels in conditioned medium of Act-MRC-5-treated cells were determined by ELISA. * *p* < 0.05., ** *p* < 0.01, *** *p* < 0.001 vs Act-MRC-5 control. ^¶¶¶^ *p* < 0.001 vs. MRC-5 control. ^#^ *p* < 0.05 vs. free RES.

To determine the effect of L-RES on inactivating the activated fibroblast, the L-RES or free RES and LIP were treated in the Act-MRC-5activated fibroblast cells for 24 h. The expression of α-SMA was investigated using immunofluorescent staining, and IL-6 levels were determined using ELISA. Decreased expressions of α-SMA were found in both L-RES- and free-RES-treated Act-MRC-5 cells compared with the untreated Act-MRC-5 cells, whereas the LIP-treated cells were not affected (Figure 4A). This result implies that both free RES and L-RES were sufficient to inactivate the activated fibroblast. The IL-6 levels in cells treated with L-RES, free RES, and LIP were 395.4 ± 7.0 pg/mL, 573.7 ± 7.6 pg/mL, and 939.5 ± 2.7 pg/mL, respectively; in contrast, the IL-6 levels in untreated Act-MRC-5 cells were 668.9 ± 0.3 pg/mL (Figure 4B). IL-6 levels were decreased in L-RES-treated Act-MRC-5 cells compared with untreated Act-MRC-5 cells. This result suggests that L-RES could inactivate the activated fibroblast, as shown by the decreased expression of α-SMA, activated fibroblast marker, and IL-6 production.

### 3.6. Effect of L-RES-Treated Act-MRC-5 Cells on Cell Migration and Invasion of CRC Cells in Co-Culture Model

**Figure 5:**
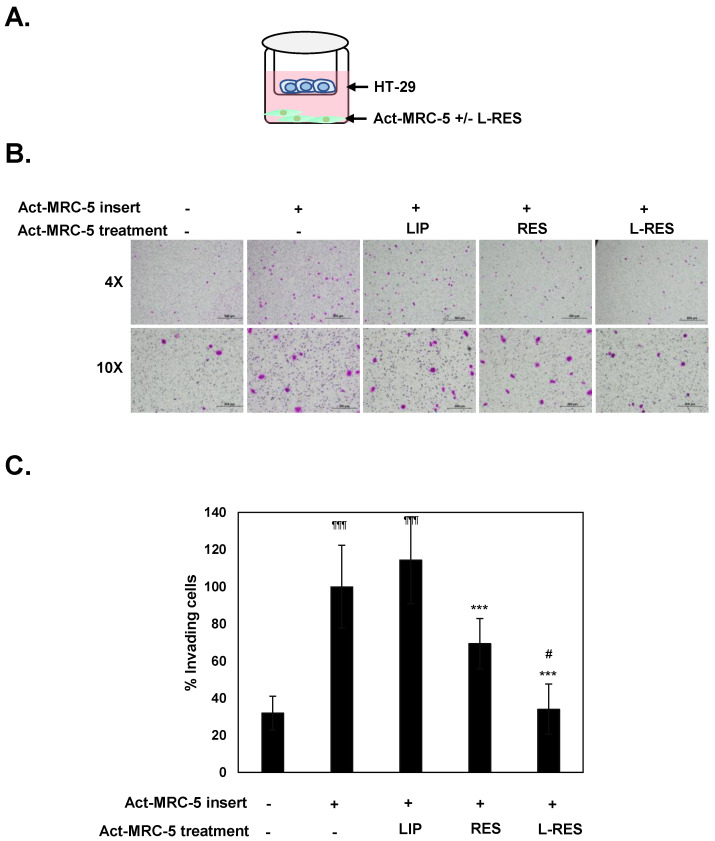
L-RES treatments diminish the activated fibroblasts promote cancer cell invasion. The HT-29 cells were co-cultured with the activated fibroblast; Act-MRC-5, which was treated with RES; LIP; or L-RES. Cell migration or cell invasion was determined using Boyden chamber assay for 24 h. (**A**) Co-culture between HT-29 and Act-MRC-5 with treatments. (**B**) The invaded cells were captured using an inverted microscope with five fields/well, in triplicated well at 10× magnification. (**C**) Percentages of invaded cells were presented (untreated Act-MRC-5 control cells = 100%). The data are means ± SD from the representative experiments. *** *p* < 0.05 vs. Act-MRC-5 control. ^¶¶¶^ *p* < 0.001 vs. MRC-5 control. ^#^ *p* < 0.05 vs. free RES.

To investigate the effect of inactivated Act-MRC-5 cells using L-RES on cell migration and cell invasion of CRC cells, we used a co-culture model in the transwell plate (Figure 5A). Co-culture of CRC cells and the Act-MRC-5 activated fibroblast enhances cell invasion of CRC cells 18.33 ± 3.8-folds, compared with MRC-5 control cells (Figure 5B,C). The cell invasion ability of CRC cells in the co-culture model treated with L-RES, free RES, and LIP were 34.5 ± 6.8%, 78.2 ± 15.6%, and 105.0 ± 3.82%, respectively. These results confirmed that L-RES-treated Act-MRC-5 more strongly inhibited cell invasion of CRC cells than free RES. Previous studies have reported the inactivation of activated fibroblasts, NIH3T3 cells, or primary CAF using gold-core silver-shell hybrid nanoparticles, which decreased cell migration and cell invasion of breast cancer cells in co-culture condition [49]. Our study is the first to report resveratrol-loaded nanoparticles can disrupt crosstalk communication between cancer cells and activated fibroblasts.

### 3.7. Effect of L-RES-Treated Act-MRC-5 Cells on Tumor Spheroid Invasion of CRC Cells

**Figure 6:**
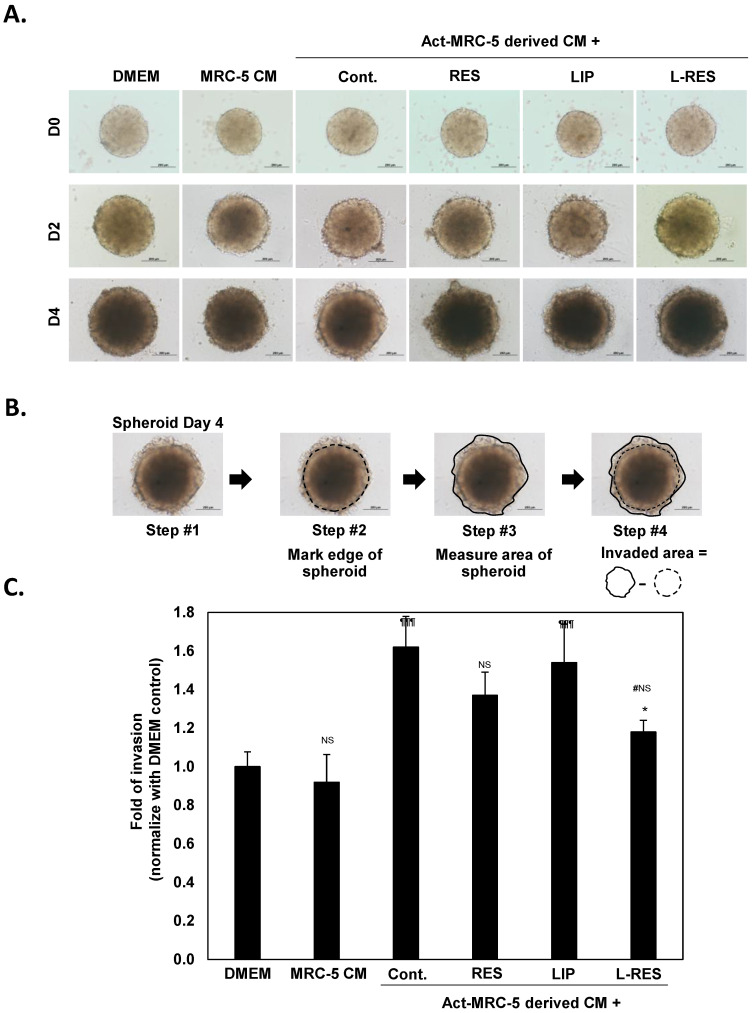
L-RES treatment alleviated the cancer cell invasion promoting activity of activated fibroblasts in 3D tumor spheroid invasion assay. The HT-29 tumor spheroids were established. Three-day-old spheroids were embedded in Matrigel for exploring the invasion ability of the tumor spheroid. Then, RES-, LIP-, or L-RES-treated Act-MRC-5-derived CM was added into the spheroid for 96 h, and the invaded area was examined. (**A**) Morphology of HT-29 tumor spheroid invasion observed under inverted microscope at 10× magnification. (**B**) Calculation method of invaded area using ImageJ software. (**C**) The relative invaded area from the edge of the spheroid determined by ImageJ software (untreated control cells = 1). The data are means ± SD from the representative experiments. * *p* < 0.05 vs. Act-MRC-5 control. ^¶¶¶^ *p* < 0.001 vs. DMEM control. NS: nonsignificant.

To mimic the environment of a solid tumor, a tumor spheroid was generated for studying CRC cell invasion. The tumor spheroid was co-cultured with L-RES-treated Act-MRC-5 derived CM to test its effect on the invasiveness of CRC tumor spheroid (Figure 6). The increased invasive ability of tumor spheroid was observed when co-cultured with activated Act-MRC-5-derived CM compared with the control cells; Figure 6A,B show the tumor spheroid treated with MRC-5-derived CM (MRC-5 CM). This result confirmed the effect of activated fibroblasts on enhancing the invasiveness of CRC cells. Our results are similar to those of previous studies, which demonstrated that activated fibroblasts or CAF enhance the invasive ability of several cancer cell types [39,49,50,51]. Since we demonstrated that L-RES could inactivate the activated fibroblast, Act-MRC-5, the effect on tumor spheroid invasion was also investigated. L-RES-treated Act-MRC-5-derived CM (L-RES/Act-MRC-5 CM) showed significantly lower invasive ability in HT-29 spheroid cells compared with the Act-MRC-5 CM. In addition, compared with free-RES-treated Act-MRC-5 (free RES/Act-MRC-5), the L-RES-treated cells had a markedly stronger effect on inhibiting HT-29 spheroid invasion. This result suggests that the (L-RES/Act-MRC-5 CM) could diminish the effect of Act-MRC-5 on enhancing invasive ability of HT-29 spheroids. These results imply that L-RES could be an alternative approach for inhibiting cancer cell invasion via reprogramming the activated fibroblast in the tumor microenvironment.

### 3.8. Specificity of L-RES in Co-Culture

To investigate the specificity of L-RES to CAF-containing tumors, we used a monoculture of HT-29 tumor spheroid and co-culture of HT-29 and MRC-5 tumor spheroid to test the effect of L-RES. L-RES at various concentrations (12.5–100 µM) was directly added into the tumor spheroid for 48 h (Figure S2), and the spheroid’s morphology was observed under the microscope. The results revealed that the HT-29 monoculture spheroid was deformed at concentrations of 50–100 µM, while co-culture was found at concentrations of 12.5–100 µM (Figure S2). These results suggest that L-RES was more specific in inhibiting spheroid growth of tumors containing CAF or activated fibroblasts, which mimic the tumor microenvironment of solid tumors.

### 3.9. Effect of L-RES on Drug Sensitivity of 3D Tumor Spheroid (Monoculture and Co-Culture)

**Figure 7:**
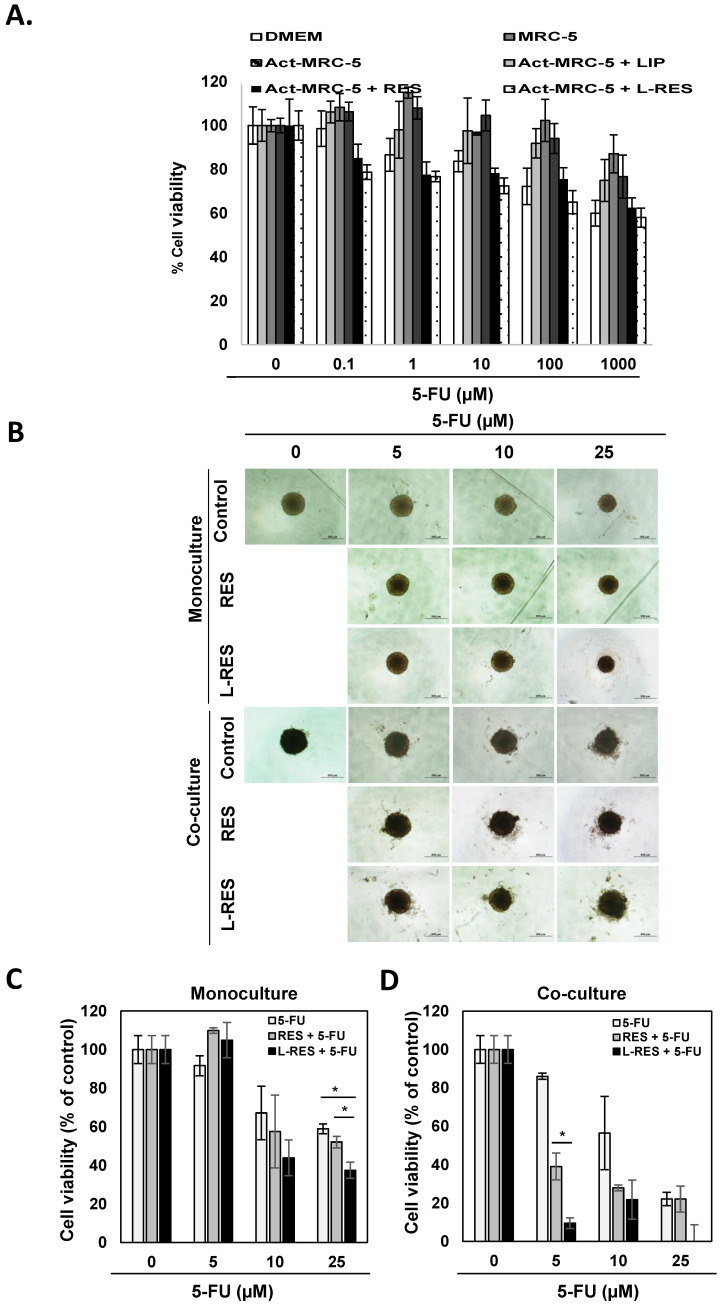
L-RES treatment sensitizes CRC cells to chemotherapeutic drugs when co-cultured with activated fibroblasts. (**A**) HT-29 cell monolayers were exposed to conditioned medium-derived Act-MRC-5 treated cells (treated with RES, LIP, or L-RES (25 µM)) and combined with 5-FU at various concentrations (0–1,000 µM). Cell viability of HT-29 was analyzed using MTT assay (untreated control cells = 100%). The data are means ± SD from the representative experiments. (**B**) The tumor spheroids of monoculture HT-29 tumor spheroid (upper panel) or co-cultured HT-29 and MRC-5 cells (lower panel) were established. Three-day-old spheroids were treated with RES or L-RES at a concentration of 25 µM with or without 5-FU at various concentrations (0–25 µM) for 48 h. The morphology of the tumor spheroid was observed under an inverted microscope at 4× magnification. Scale bar = 500 µm. (**C**) Cell viability of monoculture tumor spheroid or (**D**) co-culture tumor spheroid was determined using ATPlite luminescence assay. The data are means ± SD from the representative experiments. * *p* < 0.05, NS = nonsignificant.

The roles that CAF and activated fibroblasts play in enhancing drug resistance of cancer cells has been documented [6]. The effect of activated fibroblasts on promoting the drug resistance of CRC has also been demonstrated in a monolayer culture model. In addition, the action of activated fibroblasts on promoting drug resistance of CRC was attenuated by L-RES treatment (Figure 7A). Act-MRC-5-derived conditioned medium with different treatments (L-RES, free RES, or LIP) was used to culture CRC cells with or without 5-FU (0–1,000 µM). Compared with culturing CRC cells in DMEM, the Act-MRC-5-derived CM promoted CRC cell growth. However, the growth-promoting effect was not found in MRC-5-derived CM, which was remarkably resistant to 5-FU compared with the control DMEM group. This phenomenon was inhibited by RES- and L-RES-treated Act-MRC-5-derived CM. However, there was no significant difference between free RES and L-RES treatment, possibly because both free RES and L-RES at this concentration could inhibit the characteristics of activated fibroblasts (Figure 7A).

To confirm this phenomenon, the CRC tumor spheroid was established for testing in both monoculture and co-culture conditions and treated with free RES or 25 µM of L-RES in combination with 5-FU at concentrations of 5–25 µM. In the monoculture of HT-29 tumor spheroid, L-RES-treated spheroid in combination with 5-FU treatment showed obviously smaller spheroid size in a dose-dependent manner compared with free RES (Figure 7B, upper panel). This result implies that L-RES enhanced 5-FU sensitivity of CRC cells. For 3D tumor spheroid co-culture, HT-29 cells were cultured with activated fibroblasts, Act-MRC-5. In addition, for the L-RES-treated spheroid combined with 5-FU at 5 µM, deformity of the spheroid was observed, but in free resveratrol, the morphology was unchanged (Figure 7B, lower panel). Cell viability of the tumor spheroid was evaluated using ATPlite luminescence assay. Cell viability of monoculture HT-29 tumor spheroid of 5-FU-treated cells was decreased in a dose-dependent manner (Figure 7C). Cell viability at 5, 10, and 25 µM was 91.7 ± 5.2%, 67.2 ± 13.9%, and 58.9 ± 2.6%, respectively. At 5-FU concentrations of 25 µM, cell viability of monoculture HT-29 tumor spheroid combined with RES and L-RES was 52.1 ± 3.0% and 37.5 ± 4.2%, respectively. This result revealed that L-RES enhanced 5-FU sensitizing of HT-29 spheroid as observed in treatment of 5-FU at concentration of 25 µM. L-RES significantly enhanced the growth-inhibiting effect of 5-FU in the HT-29 spheroid monoculture model. In co-cultured tumor spheroids, cell viability of 5, 10, and 25 µM was 86.0 ± 1.6%, 56.4 ± 19.1%, and 22.1 ± 3.5%, respectively (Figure 7D). At 5-FU concentrations of 5 µM, cell viability of co-cultured tumor spheroids combined with RES and L-RES was 39.1 ± 7.1% and 9.5 ± 2.8%, respectively. Cell viability of co-cultured tumor spheroid was significantly inhibited in co-treatment with L-RES and 5-FU at low concentration of 5-FU at 5 µM. This result implies that L-RES enhanced the sensitizing effect of 5-FU in co-cultured tumor spheroid. L-RES had a significantly stronger effect than RES. This result suggests that the co-cultured tumor spheroid treated with L-RES showed effects more specific to fibroblasts and had more potent effects than those of the monoculture spheroid. This result implies that L-RES could enhance 5-FU sensitivity of 3D tumor spheroid co-culture.

### 3.10. L-RES Supressed the Expressions of EMT-Associated Genes and L1CAM 

To identify the mechanism underlying diminished activation of fibroblast by L-Res suppressed cell invasion of CRC cells, the EMT relating genes; snail-1, slug, vimentin, E-cadherin, N-cadherin, and L1CAM, which are involved in CRC cancer cell migration and invasion process [52,53], were determined. Epithelial to mesenchymal transition is a crucial process in metastasis of cancer which confers metastatic properties upon cancer cells by enhancing migration and invasion. Several molecules are involved in this process, such as adhesion molecules, MMP, and EMT regulatory proteins, etc. The loss of epithelial cell markers and E-cadherin, and the gain of mesenchymal cell markers, including vimentin, snail1, slug, and N-cadherin, are common features of EMT [52,54]. CAF promotes cancer metastasis in lung, oral, bladder cancer, and CRC via enhancing EMT [55,56,57,58]. 

**Figure 8:**
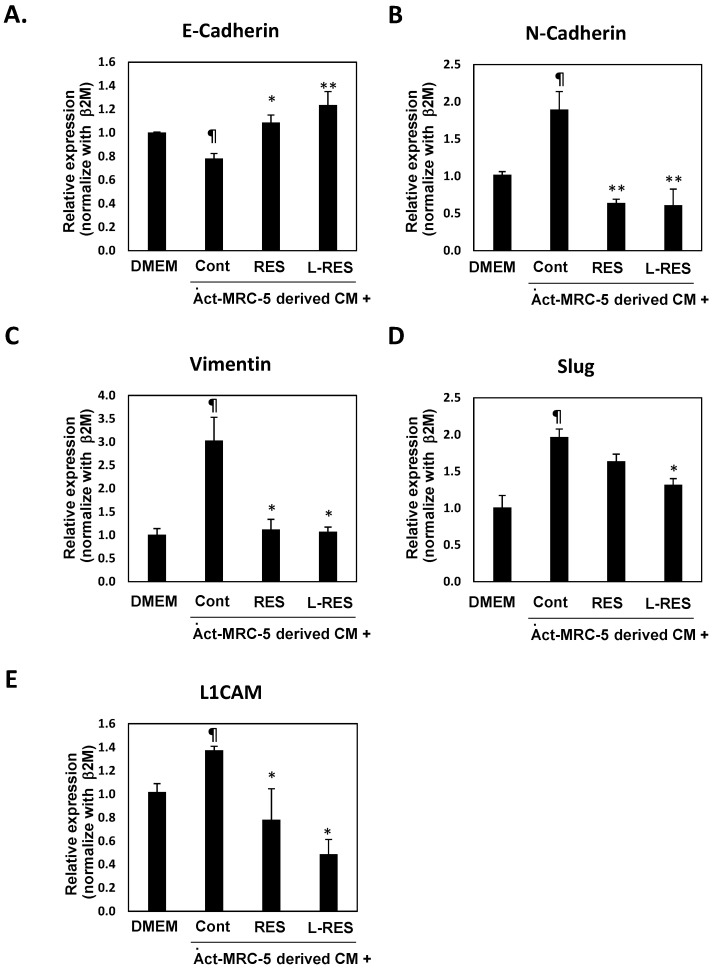
Activated-fibroblast-induce EMT related genes in CRC cells and abrogated by L-RES treatment. The expressions of EMT and L1CAM related to cell migration and invasion in CRC cells, namely (**A**) E-cadherin, (**B**) N-Cadherin, (**C**) Vimentin, and (**D**) Slug, were determined in HT-29 cells treated with conditioned medium derived RES or L-RES pretreated Act-MRC-5 using quantitative real-time PCR. Relative mRNA expression was calculated (untreated control cells = 1). The data are means ± SD from the representative experiments. * *p* < 0.05, ** *p* < 0.01, *** *p* < 0.001 vs Act-MRC-5 control. ^¶^ *p* < 0.05 vs DMEM control.

Quantitative real time PCR analysis of E-cadherin, N-cadherin, Vimentin, and Slug) indicated that Act-MRC-5 CM treated HT-29 cells expressed lower levels of E-cadherin and higher expressions of N-cadherin, Vimentin, and Slug than their untreated control cells, as shown in Figure 8A-D. This result confirmed that activated fibroblast enhance EMT process in CRC cells. This suggested that activated fibroblast induced cell invasion of CRC cells via modulating EMT process. CAF promoted metastasis of CRC cells via enhancing EMT has been reported [58]. Interestingly, Act-MRC-5-RES and Act-MRC-5 L-RES CM treated HT-29 cells expressed higher levels of E-cadherin and lower expressions of N-cadherin, Vimentin and Slug than their Act-MRC-5 CM treated cells, Figure 8A-D. These results implied that free RES and L-RES could inhibit EMT process in CRC cells treated with activated fibroblast CM. In CRC cells, resveratrol inhibits metastasis via suppressing EMT in LoVo cells [42]. In conclusion, the current study demonstrated that diminished activation of fibroblast by L-RES suppressed cell invasion of CRC cells via inhibiting EMT process. Crosstalk of activated fibroblast and CRC cells in promoting CRC invasion via enhancing EMT activity was interrupted by L-RES which led to reducing cell invasion ability by suppressing EMT process.

Moreover, expression of L1CAM, which is a metastatic related gene, involved in CRC cell migration and invasion process [53] was observed. The result indicated that L1CAM was induced in Act-MRC-5 CM treated HT-29 cells as shown in Figure 8E. It should be noted that Act-MRC-5-RES and Act-MRC-5 L-RES CM treated HT-29 cells expressed lower levels of L1CAM expressions than their Act-MRC-5 CM treated cells, as shown in Figure 8E. These results implied that free RES and L-RES could inhibit expression of L1CAM in CRC cells treated with activated fibroblast CM. This result confirmed that L-RES inhibits cell invasion of CRC cell via regulating L1CAM, partially.

## 4. Conclusions

L-RES had enhanced antitumor activity and diminished activation of activated fibroblasts into inactive fibroblasts to inhibit the CRC-promoting effects (i.e., cell migration, cell invasion, and drug resistance) of activated fibroblasts. Moreover, L-RES at the concentration which inhibited fibroblasts was also specific to activated fibroblasts in the condition mimicking the tumor microenvironment. Treating L-RES is successful to modulate CAF resulting in enhancing drug sensitivity of CRC to 5-FU according to the gene expression quantitation. Taken together, these findings indicate that L-RES could potentially be used as an alternative agent for enhancing antitumor activity via modulating the activated fibroblast in the tumor microenvironment of CRC.

## Figures and Tables

**Table 1:** Physicochemical properties of resveratrol loaded liposome liposomes. The hydrodynamic size, polydispersity index (PDI), and zeta potential of liposomes were measured by the dynamic light scattering. Encapsulation efficiency was determined using HPLC.

Samples	Size (nm)	Zeta Potential (mV)	PDI	%EE of RES
LIP	179.77 ± 5.67	−11.33 ± 0.38	0.11 ± 0.03	-
L-RES	167.30 ± 2.79	−10.90 ± 0.46	0.20 ± 0.02	98.20

## Data Availability

The data are available on reasonable request from the corresponding author.

## Supplementary Materials

The following supporting information can be downloaded at: https://www.mdpi.com/article/10.3390/nano13010107/s1, Figure S1: CRC-cell-derived conditioned medium used to induce activated fibroblasts, Figure S2: Activated-fibroblast-containing tumor spheroid was more sensitized to L-RES.
