# Supplementary material for: Resveratrol Loaded Liposomes Disrupt Cancer Associated Fibroblast Communications within the Tumor Microenvironment to Inhibit Colorectal Cancer Aggressiveness
Source: Nanomaterials (Basel). 2022 Dec 25;13(1):107. doi: 10.3390/nano13010107 (PMC9824711; doi:10.3390/nano13010107)
Supplement: Supplementary file 1 [file nanomaterials-13-00107-s001.zip › nanomaterials-2102621-supplementary.pdf]

Supplementary file

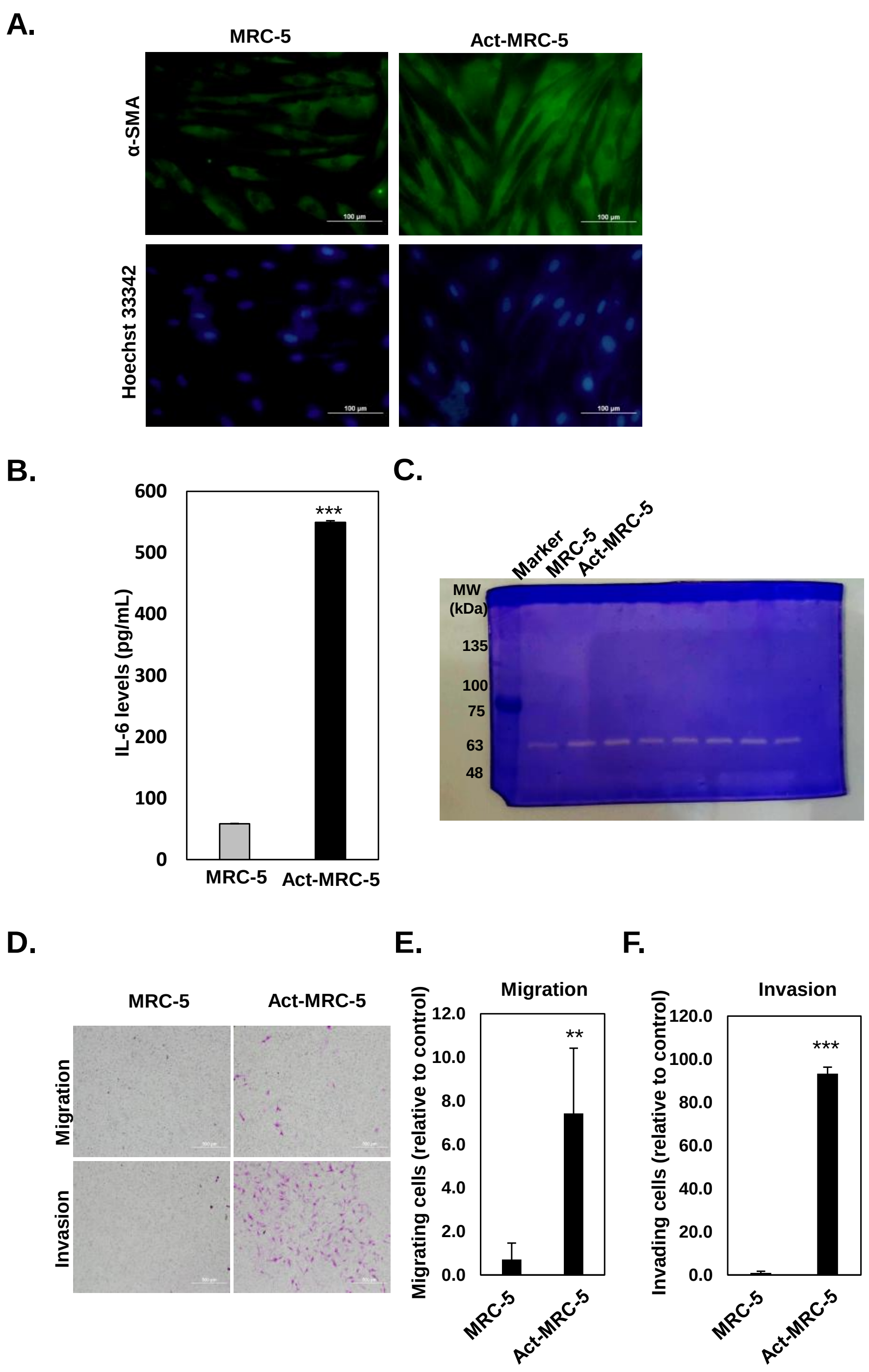

Figure. S1. CRC-cell-derived conditioned medium used to induce activated fibroblasts. Normal human fibroblast cells (MRC-5 cells) were treated with HT-29 cells derived conditioned medium for 24 h. (A) Expression of α-SMA in the MRC-5 normal human fibroblast cells was determined by immunocytofluorescent labeling. (B) IL-6 levels in

conditioned medium of MRC-5-treated cells analyzed by ELISA. (C) MMPs 2/9 activities (D) Cell migration and cell invasion abilities of MRC-5-treated cells analyzed by Boyden chamber assay. The migrated and invaded cells were captured using an inverted microscope with 5 fields/well, in triplicated well at 10× magnification. Relative of migrated cells (E) and invaded cells (F) are presented (untreated control cells = 100%). The data are means ± SD from the representative experiments. ** $P < 0.01$.,*** $P < 0.001$.

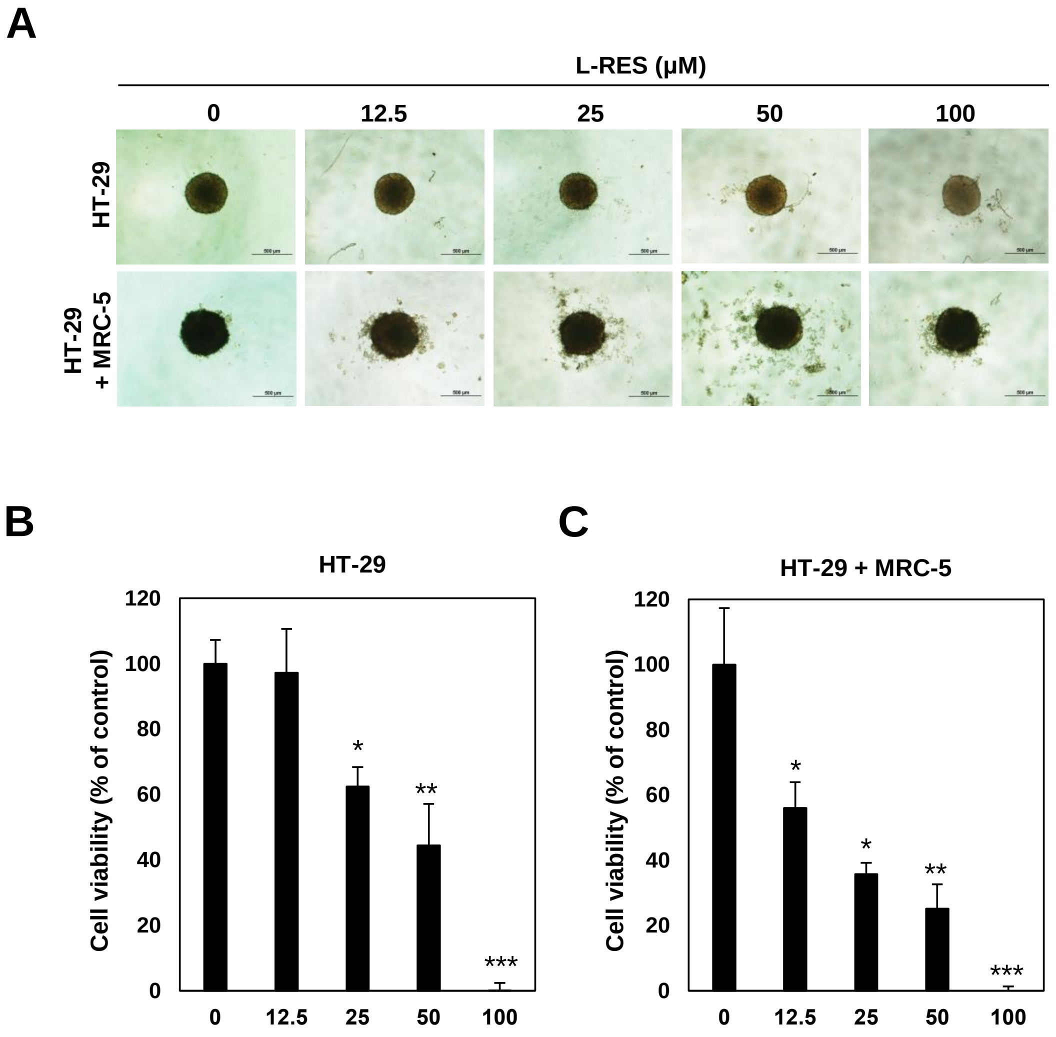

Figure. S2. Activated-fibroblast-containing tumor spheroid was more sensitized to L-RES. The tumor spheroids of monoculture of HT-29 tumor spheroid (upper panel) or co-cultured HT-29 and MRC-5 cells (lower panel) were established. Three-day-old spheroids were treated with L-RES at various concentrations (12.5–100 µM) for 48 h. (A) The morphology of the tumor spheroid was observed under inverted microscope at 4× magnification. Scale bar = 500 µm. Dot boxes emphasize the minimum concentration in which spheroid deformity was observed. (B) Quantitative analysis of cell viability of monoculture tumor spheroid or (C) Quantitative analysis of co-cultured tumor spheroid was determined using ATPlite luminescence assay. The data are means ± SD from the representative experiments. *P < 0.05., **P < 0.01, ***P < 0.001.
